# Ecotoxicological Effects of Psychoactive Pharmaceuticals in *Lemna minor*: Phytoremediation Potential and Mixture Risk Assessment

**DOI:** 10.3390/toxics14050420

**Published:** 2026-05-12

**Authors:** Nicole Geraldine de Paula Marques Witt, Daiana Castro Barros, Bruna Franciscon de Oliveira, Breno Lourenzzo Salgado Guimarães, Diego Dias Sudul, Philippe Juneau, Marcelo Pedrosa Gomes

**Affiliations:** 1 Laboratório de Fisiologia de Plantas sob Estresse, Departamento de Botânica, Setor de Ciências Biológicas, Universidade Federal do Paraná, Avenida Coronel Francisco H. dos Santos, 100, Centro Politécnico Jardim das Américas, Curitiba 81531-980, Paraná, Brazil; nicabio@ufpr.br (N.G.d.P.M.W.); daianabarros@ufpr.br (D.C.B.); 2 Programa de Pós-Graduação em Ecologia e Conservação, Setor de Ciências Biológicas, Universidade Federal do Paraná, Avenida Coronel Francisco H. dos Santos, 100, Centro Politécnico Jardim das Américas, Curitiba 81531-980, Paraná, Brazil; brunafranciscon@ufpr.br (B.F.d.O.); breno.guimaraes@ufpr.br (B.L.S.G.); diegodias1405@gmail.com (D.D.S.); 3 Ecotoxicology of Aquatic Microorganisms Laboratory, EcotoQ, TOXEN, GRIL, Department of Biological Sciences, Université du Québec à Montréal, Montréal, Succ. Centre-Ville, Montréal, QC H3C 3P8, Canada; juneau.philippe@uqam.ca

**Keywords:** pharmaceuticals, ecotoxicology, phytoremediation, mixture risk assessment, species sensitivity distribution

## Abstract

Background: The increasing consumption of psychoactive pharmaceuticals has led to their continuous release into aquatic environments. Methods: This study assessed the ecotoxicological responses, phytoremediation capacity, and ecological risk of seven psychoactive pharmaceuticals—citalopram (CIT), sertraline (SER), fluoxetine (FLU), alprazolam (ALP), clonazepam (CLO), risperidone (RIS), and topiramate (TOP)—using *Lemna minor* under controlled exposure conditions. Plants were exposed to a concentration gradient, and physiological endpoints, including relative growth rate, chlorophyll content, and maximum photosystem II efficiency (Fv/Fm), were evaluated alongside compound removal and abiotic degradation. Results: Dose–response modeling revealed substantial variability in toxicity, with TOP (EC_50_ = 74.11 ng L^−1^), CLO (104.8 ng L^−1^), and RIS (138.5 ng L^−1^) exhibiting the highest potency, whereas FLU (1751 ng L^−1^), CIT (89,941 ng L^−1^), and ALP (465,351 ng L^−1^) were less toxic. Relative growth rate was the most sensitive endpoint. Mixture exposure did not result in additional toxicity compared to the most responsive individual compounds. Abiotic degradation was negligible for most compounds (<3%), except for SER (~42%) and FLU (~22%). In contrast, *L. minor* achieved net removal efficiencies of up to 81%, although reductions occurred under mixed conditions. Probabilistic risk assessment indicated a high ecological risk (msPAFtotal = 1.0), with RIS as the dominant contributor.

## 1. Introduction

The continuous release of psychoactive pharmaceuticals into aquatic systems is a growing concern in environmental ecotoxicology. These compounds are primarily introduced through human consumption and are only partially removed during conventional wastewater treatment, resulting in their widespread occurrence in surface waters [1,2]. Reported environmental concentrations vary considerably among compounds and locations but frequently occur within the ng L^−1^ to low µg L^−1^ ranges. Selective serotonin reuptake inhibitors (SSRIs), for example, have been detected at comparatively elevated levels, with citalopram reaching up to 76,000 ng L^−1^, fluoxetine ranging from 12 to 1400 ng L^−1^, and sertraline typically occurring between 30 and 164 ng L^−1^ [3,4,5]. Other psychotropic compounds, including the anxiolytics alprazolam (up to 5900 ng L^−1^) and clonazepam (up to 100 ng L^−1^), as well as the antipsychotic risperidone (~0.34 ng L^−1^) and the anticonvulsant topiramate (up to 145 ng L^−1^), have also been reported, reflecting a chemically diverse and persistent contaminant mixture in aquatic environments [4,6].

The ecological relevance of these concentrations is supported by a growing body of evidence demonstrating their adverse effects on aquatic organisms. Neuroactive pharmaceuticals act on highly conserved signaling pathways in many organisms, and even at sublethal levels, they can induce significant alterations in the behavior, physiology, and development of non-target aquatic species. For instance, citalopram has been shown to impair locomotion and survival in *Daphnia magna* and inhibit the growth of freshwater microalgae [7,8]. Sertraline, often identified as one of the most toxic selective SSRIs in aquatic systems, induces neurobehavioral disturbances, including reduced locomotor activity and altered predator responses, as well as endocrine disruption and oxidative stress [9,10,11]. Similarly, fluoxetine has been associated with reproductive and behavioral alterations in fish species, such as *Danio rerio* and *Poecilia reticulata*, even at concentrations below 1 µg L^−1^ [12]. Benzodiazepines and antipsychotics further contribute to this scenario, with compounds such as clonazepam and risperidone inducing neurological, developmental, and behavioral effects that may compromise survival and ecological interactions [13,14].

These findings raise concerns that extend beyond individual-level toxicity. Behavioral disruption, altered predator–prey dynamics, and reproductive impairment may collectively translate into population-level effects and ecosystem instability. Importantly, exposure to natural systems is not limited to single compounds but occurs as complex mixtures, in which interactions may lead to additive or synergistic effects; however, these responses vary across environmental contexts [15]. However, mixture toxicity remains insufficiently addressed in current ecotoxicological frameworks, particularly for neuroactive compounds.

Given this scenario, there is a growing need for sustainable strategies to mitigate the environmental dispersion of psychoactive pharmaceuticals in the aquatic environment. Nature-based solutions, particularly those involving aquatic plants, have emerged as promising alternatives owing to their capacity to remove, transform, or stabilize contaminants while providing additional ecosystem services [16]. In this context, phytoremediation offers a low-cost, ecologically compatible approach that can complement conventional treatment systems [16]. Among aquatic macrophytes, *Lemna minor* has been extensively used as a model organism in ecotoxicological studies because of its rapid growth, high sensitivity to environmental stressors, and standardized use in regulatory assays [17]. In addition to its role as a bioindicator, *L. minor* removes nutrients, trace elements, and organic pollutants from contaminated water [18,19]. Recent studies suggest that this species may also contribute to the attenuation of pharmaceutical contaminants through uptake, transformation, and interaction with associated microbiota, although the relative contributions of these processes remain unresolved in most experimental systems [17,20]. Despite the growing interest in plant-based remediation, the ecotoxicological responses of aquatic phototrophs to psychoactive pharmaceuticals remain unexplored. Most available studies focus on animal models, while the effects of these compounds on primary producers—and their potential role as both targets and mitigators of contamination—remain insufficiently characterized. This gap is particularly relevant given the central role of primary producers in aquatic food webs and their direct involvement in contaminant cycling.

In this context, the present study investigated seven psychoactive pharmaceuticals frequently detected in aquatic environments—citalopram, sertraline, fluoxetine, alprazolam, clonazepam, risperidone, and topiramate—selected based on their widespread use, environmental persistence, and biological activity [5,21]. This study aimed to (i) evaluate and compare the ecotoxicological effects of environmentally relevant concentrations of these compounds, individually and in mixtures; (ii) assess the capacity of *L. minor* to contribute to their removal from aqueous systems; and (iii) perform an environmental risk assessment to estimate the potential ecological implications associated with their occurrence in aquatic ecosystems. We hypothesized that these psychoactive pharmaceuticals, even at low and environmentally realistic concentrations, induce measurable physiological and growth-related responses in *L. minor*, reflecting their potential interaction with conserved biochemical pathways, leading to a trade-off between the toxicity and phytoremediation efficiency of the plant. Furthermore, we expect that mixture exposure may result in non-additive effects, depending on the compound interactions.

## 2. Materials and Methods

### 2.1. Chemicals and Reagents

All pharmaceutical standards (citalopram, sertraline, fluoxetine, alprazolam, clonazepam, risperidone, and topiramate) were of analytical grade and purchased from Sigma-Aldrich (São Paulo, Brazil). Stock solutions were prepared according to the solubility of each compound. Owing to their lipophilic nature, alprazolam, risperidone, and citalopram were initially dissolved in dimethyl sulfoxide (DMSO) and then diluted in the CHU10 culture medium. The remaining compounds were directly dissolved in the CHU10 medium. The final concentration of DMSO in all treatments did not exceed 0.1% (*v*/*v*), and no significant differences were observed between the solvent controls and control treatments (Table S1).

### 2.2. Plant Material and Culture Conditions

*Lemna minor* L. was used as the model organism in this study. Plants were maintained in sterile CHU10 medium (pH 7.4) [22] under controlled conditions prior to exposure. Cultures were maintained in growth chambers (BOD) at 22 ± 2 °C under a 12 h light/12 h dark photoperiod with a photosynthetically active radiation (PAR) of 80 ± 2 µmol photons m^−2^ s^−1^.

### 2.3. Experimental Design and Exposure Conditions

Bioassays were conducted in 50 mL Erlenmeyer flasks containing 30 mL of culture medium and 0.2 g of fresh plant weight, under the same growth conditions as explained in the previous section, for a total duration of 7 days. Bioassays were conducted following the OECD guidelines for *Lemna* growth inhibition tests [23], with adaptations to incorporate multiple compounds and mixture-exposure scenarios.

A wide range of concentrations was tested for each compound to capture environmentally relevant exposure levels and enable robust concentration–response modeling. The following concentration ranges (ng L^−1^) were used: citalopram (0–10,000,000), sertraline (0–10,000), fluoxetine (0–10,000), alprazolam (0–10,000,000), clonazepam (0–10,000), risperidone (0–10,000), and topiramate (0–10,000). These ranges were defined based on the maximum measured environmental concentrations (MEC) reported in the literature: citalopram 76,000 ng L^−1^, sertraline 155 ng L^−1^, fluoxetine 706 ng L^−1^, alprazolam 5900 ng L^−1^, clonazepam 100 ng L^−1^, risperidone 50 ng L^−1^, and topiramate 145 ng L^−1^ [5,24,25,26]. These ranges were subsequently extended beyond environmentally relevant levels to allow reliable estimation of EC_10_, EC_20_, and EC_50_ values under controlled conditions.

In addition to single-compound exposures, a mixture treatment was established using MEC values, representing the worst-case environmentally relevant mixture scenario: citalopram (76,000 ng L^−1^), sertraline (155 ng L^−1^), fluoxetine (706 ng L^−1^), alprazolam (5900 ng L^−1^), clonazepam (100 ng L^−1^), risperidone (50 ng L^−1^), and topiramate (145 ng L^−1^). To account for abiotic dissipation processes, parallel controls without plants were maintained under identical experimental conditions for each treatment.

### 2.4. Ecotoxicological Endpoints

The relative growth rate (RGR) was determined based on fresh weight measurements at the beginning (day 0) and end (day 7) of exposure as follows [23]:

$$RGR=\frac{ln(W_{t})-ln(W_{0})}{t}$$

where $W_{0}$ and $W_{t}\;$ represent the initial and final fresh weights, respectively, and t is the exposure time (days). Photosynthetic performance was assessed using a Leaf-State Analyzer (LSA-2050, Walz, Eiffeltrich, Germany). Plants were dark-acclimated for 15 min before measurement. Three independent measurements were obtained for each flask from the different plants. The maximum photosystem II efficiency (Fv/Fm) was calculated as follows [27]:

$$\frac{F_{v}}{F_{m}}=\frac{F_{m}-F_{0}}{F_{m}}$$

Chlorophyll concentration was estimated using the SPAD values obtained with a portable chlorophyll meter (ClorofiLOG CFL103, Falker, Porto Alegre, Brazil). Measurements were performed on intact fronds under the same conditions as those used for the fluorescence analyses.

### 2.5. Pharmaceutical Quantification and Analytical Procedures

Pharmaceutical concentrations in the exposure media were determined at the beginning (T_0_) and end (T_7_) of the bioassays to evaluate compound stability, abiotic dissipation and plant-mediated removal. Analyses were performed using ultra-performance liquid chromatography coupled with tandem mass spectrometry (UPLC–MS/MS), following a validated multi-residue method adapted from previously established protocols [5].

Samples of the exposure medium were filtered through 0.45 µm membranes to remove suspended particles and subsequently subjected to solid-phase extraction (SPE) using Oasis HLB cartridges (6 cc, 500 mg; Waters, Milford, MA, USA), which had been previously conditioned with methanol and ultrapure water. Prior to extraction, the samples were fortified with deuterated internal standards (alprazolam-D_5_ and fluoxetine-D_5_) to correct for variations in extraction efficiency and instrumental response. After sample loading, the cartridges were dried under a gentle nitrogen stream and eluted with methanol. The extracts were concentrated under nitrogen and reconstituted in a methanol/water solution (1:9, *v*/*v*) containing 0.1% formic acid before chromatographic analysis.

Chromatographic separation was achieved using an ACQUITY UPLC I-Class system (Waters, Milford, MA, USA) equipped with an Acquity UPLC CSH C18 column (2.1 × 100 mm, 1.7 µm) maintained at 40 °C. The mobile phase consisted of (A) ultrapure water with 0.1% formic acid and (B) acetonitrile with 0.1% of formic acid. Elution was performed under gradient conditions, starting at 99% A and 1% B, maintained for 0.5 min, followed by a gradual increase to 10% B at 3 min, 40% B at 5.5 min, and 100% B at 7.5 min. This condition was maintained for 10 min before returning to the initial conditions for column re-equilibration, resulting in a total runtime of 13 min. The flow rate was set at 0.4 mL min^−1^, and the injection volume was 5 µL.

Detection was performed using a Xevo TQD triple quadrupole mass spectrometer (Waters, Milford, MA, USA) equipped with an electrospray ionization (ESI) source operating in positive ion mode. Ionization was achieved at a spray voltage of 3200 V, capillary temperature of 350 °C, source temperature of 350 °C, sheath gas pressure of 50 arbitrary units, and an auxiliary gas flow of 15 arbitrary units. Quantification was performed in multiple reaction monitoring (MRM) mode using compound-specific precursor and product ion pairs. Retention times and mass transitions were compound-specific and were used as identification criteria. For the compounds evaluated in this study, the retention times ranged from 5.26 min for citalopram to 9.14 min for risperidone. The precursor/product ion transitions were *m*/*z* 324.95 → 109.10 for citalopram, 306.20 → 148.10 for sertraline, 310.30 → 148.10 for fluoxetine, 309.30 → 205.15 for alprazolam, 316 → 270 for clonazepam, and 411 → 191 for risperidone. The collision energies varied between −13 and −35 V, depending on the compound, reflecting the differences in fragmentation behavior.

Quantification was based on external calibration using analytical standards prepared in methanol/0.1% formic acid over a concentration range of 0.1–500 ng L^−1^. Calibration curves were constructed using linear regression models and exhibited high linearity (R^2^ ≥ 0.99 for all compounds). The analytical precision, evaluated as the intraday relative standard deviation (RSD), was below 15%. The limits of detection (LOD) were determined based on signal-to-noise ratios and ranged from 0.2 to 3.0 ng L^−1^ depending on the compound, with citalopram and fluoxetine presenting LOD values of approximately 0.3 and 0.2 ng L^−1^, respectively, while higher values were observed for compounds such as clonazepam (2.5 ng L^−1^) and risperidone (3.0 ng L^−1^). The corresponding limits of quantification (LOQ) followed the conventional signal-to-noise criterion (10:1), ensuring reliable quantification at environmentally relevant concentrations.

The accuracy of the method was evaluated through recovery experiments using fortified samples, with recoveries ranging from 85% to 110% for all compounds. Matrix effects were assessed by comparing the calibration slopes obtained in solvent and matrix conditions, indicating moderate signal suppression or enhancement depending on the analyte. Quality assurance and quality control procedures included the use of blanks, spiked samples, calibration standards, and replicate analyses for each analytical batch. The instrument performance was monitored through routine calibration checks to ensure analytical stability and reproducibility throughout the study. This analytical framework provides robust and sensitive quantification of pharmaceuticals in exposure media, enabling an accurate assessment of degradation dynamics and phytoremediation efficiency.

### 2.6. Phytoremediation Efficiency

To distinguish plant-mediated removal from abiotic processes, parallel control flasks containing the same concentrations of pharmaceuticals but without plants were maintained under identical experimental conditions as the experimental flasks. Abiotic degradation (%) was calculated to quantify non-biological losses occurring during the exposure period according to the following equation:

$$Abiotic\;degradation\left(\%\right)=\frac{C_{0}-C_{t,control}}{C_{0}}\times 100$$

where C_0_ is the initial concentration and C_t_ is the concentration at time t in the plant-free control flasks. Phytoremediation efficiency (FE, %) was calculated by correcting the total removal observed in the planted systems for abiotic losses:

$$FE\left(\%\right)=\frac{{(C}_{0}-C_{t})-D_{t}}{C_{0}}\times 100$$

where C_0_ is the initial concentration, C_t_ is the concentration at time t in flasks containing plants, and D_t_ is the mean concentration loss due to abiotic processes, determined from plant-free controls. This approach allows for the quantification of net plant-associated removal, excluding losses observed in abiotic controls. It should be noted that this approach quantifies net attenuation and does not allow for discrimination among uptake, surface adsorption, metabolic transformation, or microbiota-mediated processes.

### 2.7. Ecological Risk Assessment

Ecological risk was assessed using both a conventional quotient-based approach and a probabilistic species sensitivity distribution (SSD) framework. The deterministic assessment followed the United States Environmental Protection Agency guidance [28], in which the predicted no-effect concentration (PNEC) was derived from toxicity thresholds reported in the literature, including no-observed-effect concentrations (NOEC), lowest-observed-effect concentrations (LOEC), median effective concentrations (EC_50_), and median lethal concentrations (LC_50_). To account for the uncertainty in interspecies extrapolation and the limited availability of chronic data for several compounds, an assessment factor of 1000 was applied to the selected toxicity endpoints. Hazard quotients (HQs) were calculated as the ratio between the MEC, obtained from literature reports of environmental occurrence, and the corresponding PNEC, according to:

$$HQ=\frac{MEC}{PNEC}$$

HQ values < 0.1, 0.1–1, 1–10, and >10 were interpreted as negligible, low, moderate, and high ecological risk, respectively.

As quotient-based approaches do not account for interspecific variations in sensitivity, risk was further evaluated using species sensitivity distributions (SSDs) following the framework described by Rico et al. [29]. This probabilistic method estimates the fraction of species that may be affected by a given exposure concentration. SSDs were constructed by assuming a log-normal distribution of toxicity data for each compound using acute and chronic ecotoxicological endpoints compiled from the literature. The dataset included representatives of cyanobacteria, algae, aquatic macrophytes, invertebrates, and vertebrates. When chronic data were unavailable but acute data were sufficiently robust, acute-to-chronic extrapolation was applied using a factor of 10 to improve taxonomic coverage and permit SSD construction. All toxicity data used for SSD construction, including species, endpoints, and literature sources, are provided in the Supplementary Material (Table S4) to ensure the transparency and reproducibility of the study.

For each compound, the parameters, corresponding to the mean and standard deviation of the log-transformed toxicity values, were estimated and used to calculate the potentially affected fraction (PAF) at a given environmental concentration as follows:

$$PAF_{x}=\Phi \left(\frac{logMEC_{x}-\mu_{x}}{\sigma_{x}}\right)$$

where $MEC_{x}\;$ is the measured environmental concentration of compound x, $\mu_{x}$, is the mean of the log-transformed toxicity distribution, $\sigma_{x}\;$ is the standard deviation, and Φ is the cumulative normal distribution function. In this framework, the PAF represents the proportion of species expected to experience effects at a specific exposure concentration.

Mixture risk was estimated using the multi-substance potentially affected fraction (msPAF) approach, which accounts for both concentration addition among chemicals sharing a toxic mode of action (TMoA) and response addition among compounds acting through different modes of action. Initially, compounds belonging to the same pharmacological group were considered to potentially share a similar TMoA because they primarily targeted similar neurophysiological systems. However, when the SSD slopes for compounds within the same group differed by more than 10%, they were treated as separate TMoAs. Within each TMoA group, hazard units (HU) were calculated to normalize the differences in compound potency based on the ratio between the MEC and SSD location parameters for the corresponding compound. The combined effect for each TMoA was then estimated by assuming a concentration addition as follows:

$$msPAF_{TMoA}=\Phi \left(\frac{HU_{TMoA}}{\sigma_{TMoA}}\right)$$

The total mixture risk across TMoAs was subsequently estimated by assuming a response addition:

$$msPAF_{Total}=1-\prod_{i=1}^{n}(1-msPAF_{TMoA,i})$$

where n is the number of distinct toxic mode-of-action groups. Both single-compound PAF and total msPAF were interpreted as fractions of species that were potentially affected by the examined exposure scenario. Following Posthuma et al. [30], a hazardous concentration affecting 5% of species was considered a threshold for ecologically unacceptable effects, whereas values between 1% and 5% were interpreted as indicating a low-to-moderate ecological impact.

### 2.8. Statistical Analysis

All biological data are expressed as mean ± standard deviation based on independent replicates (n = 3). Data normality and homoscedasticity were assessed using the Shapiro–Wilk and Bartlett tests, respectively. When the assumptions were met, differences among treatments were evaluated using one-way analysis of variance (ANOVA), followed by Tukey’s post hoc test. Dunnett’s test was applied for comparisons with the control. When necessary, the data were log-transformed to meet the model assumptions. Statistical significance was set at *p* < 0.05. These analyses were performed to evaluate the treatment effects on physiological endpoints (RGR, SPAD, and Fv/Fm), including comparisons among concentrations and between single-compound and mixture exposures. All statistical analyses were performed using JMP 7.0 (SAS Institute Inc., Cary, NC, USA), except for the concentration–response modeling, which was conducted using GraphPad Prism 11 (GraphPad Software, San Diego, CA, USA). Figures were prepared using GraphPad Prism v.11.

Concentration–response relationships for RGR were modeled using non-linear regression with a four-parameter logistic model (4PL) implemented in GraphPad Prism v.11. The corresponding concentration–response curves are shown in Figure S1 (Supplementary Material). Before fitting, the RGR values were normalized relative to the mean response of the control treatment and expressed as a percentage of the control. The 4PL model included the parameters Bottom, Top, LogEC_50_, and Hill slope, all of which were initially unconstrained. When unconstrained fitting generated unstable or biologically unrealistic parameter estimates, constraints were applied, such as fixing the upper asymptote at 100% or restricting the lower asymptote to values consistent with the observed responses. The final model selection was based on the goodness of fit, parameter stability, and biological plausibility. The model performance was evaluated based on the coefficient of determination (R^2^), residual diagnostics, and parameter uncertainty, with emphasis on the normality and random distribution of residuals. Model fit quality was qualitatively classified based on R^2^ and residual behavior, with values ≥ 0.90 and normally distributed, randomly scattered residuals indicating good fit, values between 0.75 and 0.90 indicating acceptable fit, and values < 0.75 or clear violations of model assumptions indicating low robustness. From the fitted models, the EC_10_, EC_20_, and EC_50_ values were estimated along with their corresponding confidence intervals. NOEC and LOEC values were determined independently from the regression analyses using one-way ANOVA, followed by Dunnett’s post hoc test to compare each treatment with the control at α=0.05. This complementary approach allowed for a threshold-based interpretation of toxic effects, in parallel with continuous concentration–response modeling.

## 3. Results

### 3.1. Physiological Responses of Lemna minor to Increasing Concentrations of Psychoactive Pharmaceuticals

**Figure 1:**
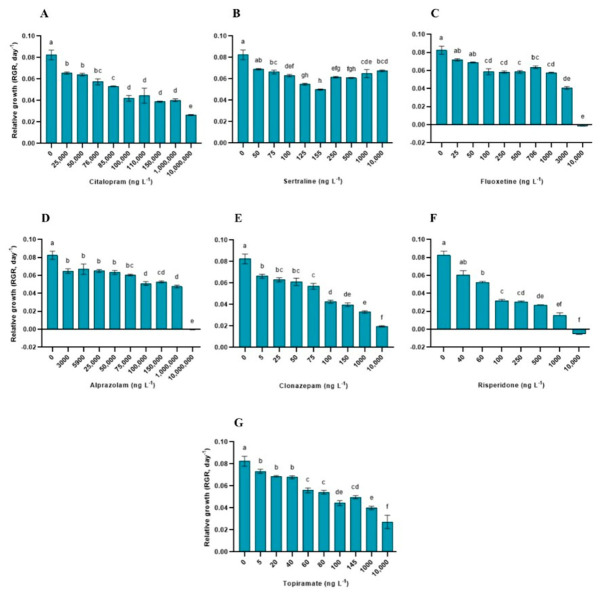
Relative growth rate (RGR) of *Lemna minor* exposed to increasing concentrations of psychoactive pharmaceuticals. Bars represent the mean ± standard deviation (n = 3). Different letters indicate significant differences among treatments (Tukey’s test, *p* < 0.05). (**A**) Citalopram (**B**) Sertraline (**C**) Fluoxetine (**D**) Alprazolam (**E**) Clonazepam (**F**) Risperidone (**G**) Topiramate.

Exposure to increasing concentrations of psychoactive pharmaceuticals significantly affected the physiological performance of *L. minor*, with distinct response patterns observed among the compounds and endpoints. RGR was significantly influenced by all the compounds tested (*p* < 0.0001 for all cases; Table S2), showing a concentration-dependent decline. For citalopram, the RGR decreased progressively from 25,000 ng L^−1^ (Figure 1). Significant reductions relative to the control were observed at ≥25,000 ng L^−1^ for citalopram, ≥75 ng L^−1^ for sertraline, ≥100 ng L^−1^ for fluoxetine, ≥3000 ng L^−1^ for alprazolam, ≥5 ng L^−1^ for clonazepam, ≥60 ng L^−1^ for risperidone, and ≥5 ng L^−1^ for topiramate. Negative RGR values were observed at the highest concentrations of fluoxetine, alprazolam, and risperidone. Across all treatments, RGR was the only endpoint that consistently responded to concentration gradients for all compounds tested.

**Figure 2:**
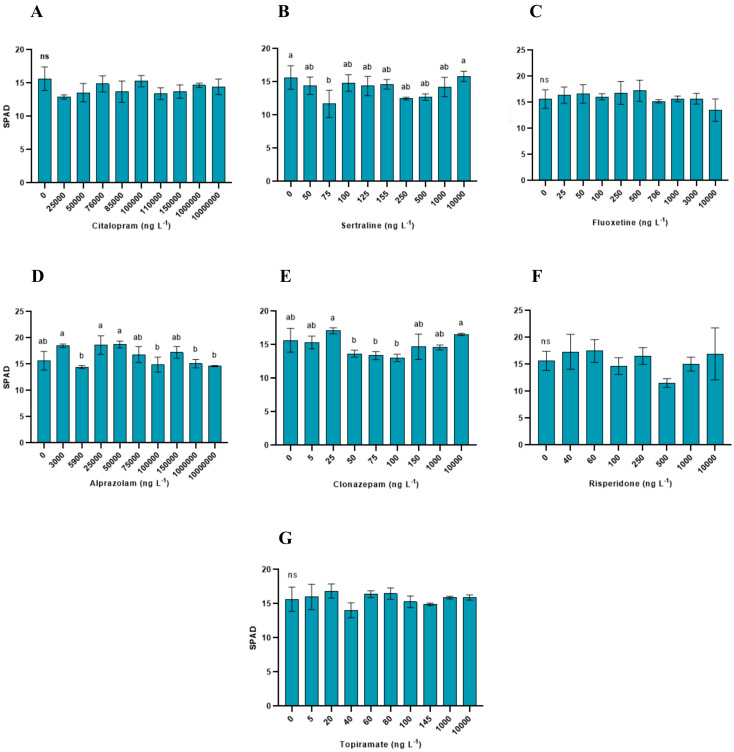
Chlorophyll concentration (SPAD values) of *Lemna minor* exposed to psychoactive pharmaceuticals. Bars represent the mean ± standard deviation (n = 3). Different letters indicate significant differences among treatments (Tukey’s test, *p* < 0.05); ns = not significant. (**A**) Citalopram (**B**) Sertraline (**C**) Fluoxetine (**D**) Alprazolam (**E**) Clonazepam (**F**) Risperidone (**G**) Topiramate.

Chlorophyll content (SPAD values) showed a more variable response depending on the treatment (Figure 2). Significant differences among the treatments were detected for sertraline (*p* = 0.0099), alprazolam (*p* = 0.0002), and clonazepam (*p* = 0.0008), whereas no significant effects were observed for citalopram (*p* = 0.098), fluoxetine (*p* = 0.2754), risperidone (*p* = 0.1221), or topiramate (*p* = 0.1102). The variations in the SPAD values were not monotonic across the concentration range.

**Figure 3:**
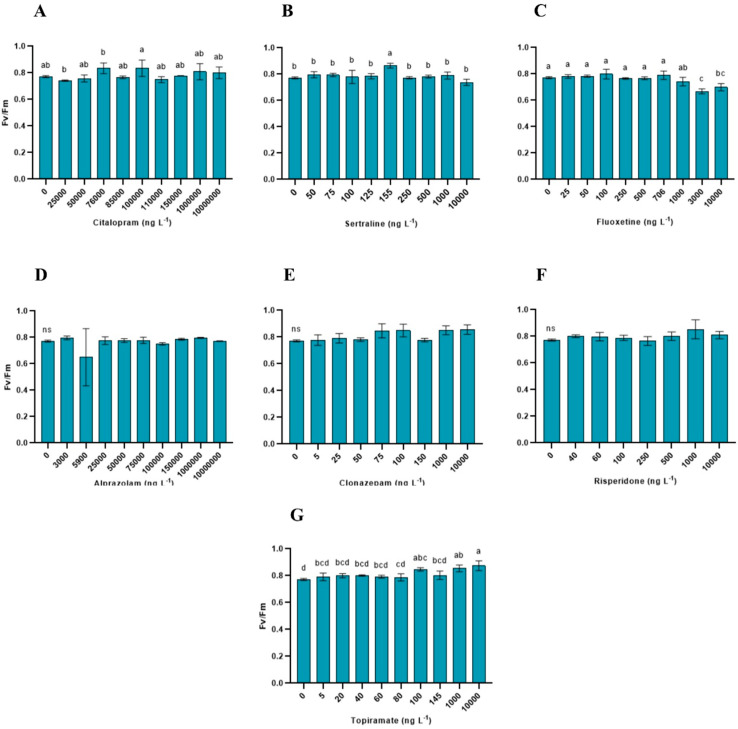
Maximum photosystem II efficiency (Fv/Fm) in *Lemna minor* exposed to psychoactive pharmaceuticals. Bars represent the mean ± standard deviation (n = 3). Different letters indicate significant differences among treatments within each compound (one-way ANOVA followed by Tukey’s test; *p* < 0.05). Non-significant responses are indicated by “ns”. (**A**) Citalopram (**B**) Sertraline (**C**) Fluoxetine (**D**) Alprazolam (**E**) Clonazepam (**F**) Risperidone (**G**) Topiramate.

Fv/Fm was significantly affected by citalopram (*p* = 0.013), sertraline (*p* = 0.0007), fluoxetine (*p* < 0.0001), clonazepam (*p* = 0.0112), and topiramate (*p* = 0.0002), whereas no significant effects were detected for alprazolam (*p* = 0.052) and risperidone (*p* = 0.1359). Changes in Fv/Fm were generally small and restricted to specific concentration ranges (Figure 3).

Concentration–response modeling based on RGR data revealed substantial variability in compound potency (Table 1 and Figure S1). Estimated EC_50_ values ranged from 74.11 ng L^−1^ for topiramate to 465,351 ng L^−1^ for alprazolam. Clonazepam and risperidone presented EC_50_ values of 104.8 and 138.5 ng L^−1^, respectively, whereas fluoxetine exhibited an EC_50_ of 1751 ng L^−1^. Citalopram showed a markedly higher EC_50_ (89,941 ng L^−1^), indicating a lower apparent toxicity within the tested range. In contrast, the EC_50_ values could not be reliably estimated for sertraline because of the absence of a well-defined sigmoidal response.

The model fit varied among the compounds, with coefficients of determination (R^2^) ranging from 0.76 to 0.92 (Table 1). The NOEC and LOEC values further reflected the differences in sensitivity among the compounds, with the lowest LOEC values observed for clonazepam (5 ng L^−1^) and topiramate (5 ng L^−1^), whereas higher thresholds were recorded for compounds such as citalopram (25,000 ng L^−1^) and alprazolam (3000 ng L^−1^). For some compounds, including citalopram and alprazolam, the NOEC values could not be determined within the tested concentration range (Table 1).

### 3.2. Physiological Responses of Lemna minor Under Environmentally Relevant Concentrations (MEC)

**Figure 4:**
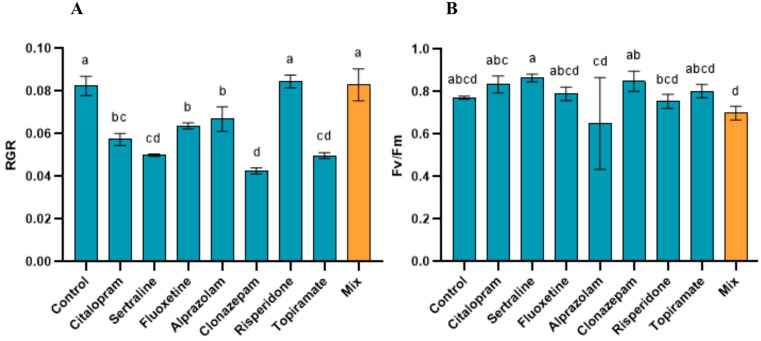
Physiological responses of *Lemna minor* exposed to environmentally relevant concentrations (MEC) of psychoactive pharmaceuticals, individually and in mixtures. (**A**) Relative growth rate (RGR) and (**B**) maximum photosystem II efficiency (Fv/Fm) of the three species. Bars represent the mean ± standard deviation (n = 3). Different letters indicate significant differences among treatments (one-way ANOVA followed by Tukey’s test, *p* < 0.05).

When plants were exposed to maximum environmentally relevant concentrations (MEC) derived from the literature, distinct physiological responses were observed for the different compounds and treatments. RGR remained the most responsive endpoint under these conditions (Figure 4A), with significant reductions detected for selected compounds. Among the tested pharmaceuticals, sertraline, clonazepam, and topiramate induced the most pronounced decrease in RGR compared to the control, whereas the remaining compounds showed either minor or non-significant effects on RGR. In contrast, Fv/Fm was not significantly affected by the individual compounds at the MEC levels (Figure 4B). No reductions relative to the control were observed in any treatment. However, sertraline exposure resulted in a significant increase in Fv/Fm compared to the control (*p* < 0.0001), indicating a deviation from the baseline photosynthetic performance, without evidence of inhibition.

When the compounds were combined in the mixture treatment, no significant reduction in Fv/Fm was observed compared to individual exposures or control (Figure 4B). Similarly, combined exposure did not result in additional reductions in RGR beyond those observed for the most responsive compounds (Figure 4A).

### 3.3. Abiotic Degradation and Phytoremediation Performance

Abiotic degradation was negligible for most compounds, remaining below 3% across all tested concentrations (Table S3). In contrast, higher degradation was observed for sertraline (41.81%) and fluoxetine (21.85%). No consistent concentration-dependent trend was observed for the abiotic degradation of each compound.

**Figure 5:**
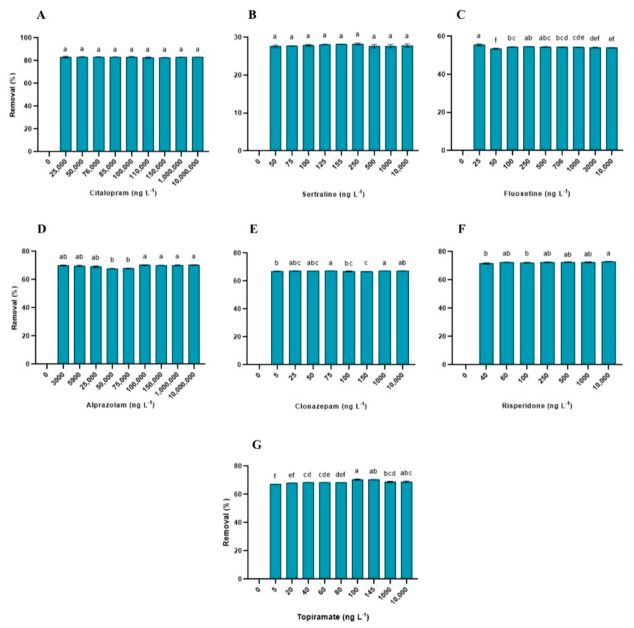
Removal efficiency (%) of psychoactive pharmaceuticals by *Lemna minor* after 7 days of exposure under isolated and mixture conditions. Bars represent the mean ± standard deviation (n = 3). Different letters indicate significant differences among treatments (one-way ANOVA followed by Tukey’s test, *p* < 0.05). (**A**) Citalopram (**B**) Sertraline (**C**) Fluoxetine (**D**) Alprazolam (**E**) Clonazepam (**F**) Risperidone (**G**) Topiramate.

In the planted systems, *L. minor* removed psychoactive pharmaceuticals with varying efficiencies (Figure 5). The highest removal efficiency was observed for citalopram (83.28 ± 0.15%), followed by risperidone (72.12 ± 0.53%), topiramate (70.41 ± 0.06%), alprazolam (69.83 ± 0.41%), clonazepam (66.93 ± 0.27%), fluoxetine (54.27 ± 0.09%), and sertraline (28.17 ± 0.02%).

Under mixture conditions, the removal efficiencies decreased for most compounds compared to isolated exposure (Table 2). Significant reductions were observed for citalopram, fluoxetine, alprazolam, clonazepam, and topiramate (Student’s *t*-test, *p* < 0.05), whereas no significant differences were detected for sertraline and risperidone.

**Table 2:** Removal efficiency (%) of psychoactive pharmaceuticals by *Lemna minor* under isolated and mixture conditions after 7 days of exposure.

Compound	Isolated	Mix	Difference
Citalopram (76,000 ng L^−1^)	83.28 ± 0.15	68.21 ± 0.15	−15.07 *
Sertraline (155 ng L^−1^)	28.17 ± 0.02	24.95 ± 0.04	−3.22
Fluoxetine (706 ng L^−1^)	54.27 ± 0.09	47.83 ± 0.21	−6.44 *
Alprazolam (5900 ng L^−1^)	69.83 ± 0.41	62.47 ± 0.58	−7.36 *
Clonazepam (100 ng L^−1^)	66.93 ± 0.27	43.12 ± 0.35	−23.81 *
Risperidone (50 ng L^−1^)	72.12 ± 0.53	66.75 ± 0.44	−5.37
Topiramate (145 ng L^−1^)	70.41 ± 0.06	51.26 ± 0.18	−19.15 *

Values represent the mean ± standard deviation (n = 3). Difference (%) indicates the variation in the removal efficiency between the isolated and mixture treatments. Negative values indicate reduced removal under mixed conditions. Asterisks (*) indicate significant differences between the isolated and mixture treatments (Student’s *t*-test, *p* < 0.05).

### 3.4. Ecological Risk Assessment and Mixture Toxicity

The ecological risk associated with the occurrence of psychoactive pharmaceuticals was evaluated using a probabilistic species sensitivity distribution (SSD) approach, considering both acute and chronic exposure scenarios (Table S4). The individual compound contributions to mixture toxicity were quantified using the toxic mode of action (μTMoA), RISKTMoA, and the resulting multi-substance potentially affected fraction (msPAF) (Table 3).

Under acute exposure conditions, all compounds contributed to the overall mixture risk, with msPAF values ranging from 0.56 for clonazepam to 0.90 for risperidone. Among the evaluated substances, risperidone exhibited the highest contribution to mixture toxicity, with a relative contribution of 67.22%, followed by citalopram (28.89%) and topiramate (19.08%). The remaining compounds showed comparatively lower contributions, although they contributed to the cumulative effect. A similar pattern was observed under chronic exposure conditions, although with increased variability in individual contributions. Risperidone was again the dominant contributor to mixture toxicity, with the highest RISKTMoA value (7.24) and a relative contribution of 91.49%. Citalopram, fluoxetine, alprazolam, and clonazepam also substantially contributed to chronic conditions, with relative contributions ranging from approximately 24% to 82%. In contrast, sertraline consistently exhibited a minor contribution to the overall risk.

Despite variability in individual compound contributions, the cumulative mixture risk remained maximal under both exposure scenarios, with msPAF_total_ values equal to 1.0 (Table 3), indicating that the combined exposure resulted in a potentially affected fraction approaching the entire species assemblage considered in the SSD analysis.

## 4. Discussion

The increasing environmental relevance of psychoactive pharmaceuticals is closely associated with their rapidly expanding global consumption, driven by the growing prevalence of mental health disorders and long-term therapeutic use [5]. Recent assessments have documented sustained increases in the prescription and environmental occurrence of antidepressants, antiepileptics, and antipsychotics, resulting in their continuous release into aquatic systems via wastewater effluents [5,21,31]. Due to their incomplete removal in conventional wastewater treatment plants (WWTPs) and high biological stability, these compounds exhibit pseudo-persistence, maintaining chronic exposure at ng–µg L^−1^ levels in surface waters [5,31,32].

In this context, the present study demonstrates that psychoactive pharmaceuticals can induce compound-specific physiological effects in *L. minor* at environmentally relevant concentrations. The observed toxicity hierarchy, with topiramate, clonazepam, and risperidone exhibiting the lowest EC_50_ values, suggests that pharmacological modes of action may interact with conserved biochemical pathways in plants. In particular, the high sensitivity to topiramate is consistent with its known inhibitory effects on carbonic anhydrase, an enzyme central to the interconversion of CO_2_ and HCO_3_^−^, and therefore to inorganic carbon acquisition and photosynthetic metabolism [33]. Disruption of this pathway can limit carbon assimilation and intracellular pH regulation, thereby affecting carbon fixation and metabolic energy balance prior to the detectable impairment of photochemical efficiency, which is consistent with the observed reduction in growth without corresponding declines in Fv/Fm.

The mechanisms of toxicity of selective SSRIs, including fluoxetine, sertraline, and citalopram, are less direct but are increasingly being documented. Although plants lack canonical serotonin reuptake systems, serotonin and related indoleamines participate in plant signaling pathways involved in growth regulation, stress responses, and redox homeostasis [34]. Experimental studies have shown that SSRIs can induce oxidative stress, alter antioxidant enzyme activity, and disrupt primary productivity in photosynthetic organisms, even at low concentrations [35,36]. These effects likely arise from cross-reactivity with conserved enzymatic systems or membrane transport processes in the plant cells.

The consistent observation that RGR was more sensitive than chlorophyll content or Fv/Fm across all compounds suggests that toxicity is not primarily associated with direct impairment of the photosystem II reaction center, but rather with downstream metabolic processes, including carbon assimilation, respiration, and cellular maintenance. Similar decoupling between growth and photochemical performance has been reported for macrophytes exposed to pharmaceuticals and other emerging contaminants [37,38]. Biomass accumulation is an integrative endpoint that captures the net balance of metabolic processes and provides a sensitive indicator of sublethal stress [23].

The persistence of growth inhibition at environmentally relevant concentrations for the selected compounds further supports their ecological significance. While the maximum photosystem II efficiency remained largely stable, reductions in RGR were observed, indicating that sublethal physiological effects can occur under realistic exposure scenarios. Interestingly, mixture exposure did not amplify the toxicity in *L. minor* and, in some cases, resulted in attenuated responses relative to individual compounds. This indicates that, under the tested conditions, the mixture effects were primarily driven by the most potent individual compounds rather than synergistic or emergent interactions. Nevertheless, the reduction in phytoremediation efficiency observed under mixed conditions suggests that interactions among compounds may affect bioavailability or plant physiological responses.

In contrast, the probabilistic risk assessment based on species sensitivity distributions (SSDs) revealed a high cumulative risk (msPAF_total_ = 1.0) for both acute and chronic exposure scenarios. Risperidone emerged as the dominant contributor to the overall risk, reflecting its high RISKTMoA values and steep position within the SSD. Importantly, this high msPAF value reflects the cumulative contribution of multiple compounds under combined exposure scenarios, rather than an enhancement of toxicity due to mixture interactions. This discrepancy between organism- and community-level responses highlights a fundamental limitation of the single-species assays. Although *L. minor* exhibited limited sensitivity to mixture effects, SSD-based approaches integrate interspecific variability and capture potential additive or synergistic interactions across taxa [39]. Consequently, they provide a more conservative estimate of ecological risk.

From an environmental fate perspective, the low abiotic degradation observed for most compounds confirms their persistence in aquatic systems. Only sertraline and fluoxetine exhibited higher abiotic degradation, which is likely associated with photochemical transformation processes [40,41]. For the remaining compounds, negligible abiotic loss indicates that biological processes are the primary drivers of environmental attenuation over short timescales.

In this regard, *L. minor* has demonstrated substantial potential for phytoremediation, achieving removal efficiencies of over 60% for several compounds. These findings are consistent with those of previous studies showing that aquatic macrophytes can effectively remove pharmaceuticals through a combination of uptake, sorption, and metabolic transformation [42]. However, the present experimental design does not allow discrimination among uptake, adsorption, metabolic transformation, or microbiota-mediated processes; therefore, removal should be interpreted as net attenuation. Additionally, the potential contribution of rhizosphere-associated microbiota to pharmaceutical attenuation cannot be excluded and remains an important source of uncertainty in the interpretation of the removal processes [38]. In addition, the removal efficiency was consistently reduced under mixture conditions, highlighting a potential limitation of phytoremediation under environmentally realistic multi-contaminant exposure scenarios. Such reductions may result from competition for transport systems, enzyme saturation, or interference among metabolic pathways, as previously observed in multi-contaminant exposure scenarios [15].

The coexistence of toxicity and removal capacity highlights an important trade-off in natural treatment systems such as wetlands. While macrophytes can contribute significantly to contaminant removal, their physiological performance may be compromised by exposure to complex mixtures. This has direct implications for the design and optimization of constructed wetlands and other plant-based treatment systems, where contaminant mixtures are the dominant exposure scenario. At the ecosystem level, a reduction in macrophyte growth may have broader implications. Aquatic macrophytes play a central role in primary production, nutrient cycling, and habitat structure [43,44]. Therefore, sustained growth impairment, even in the absence of direct PSII damage, may result in cascading ecological effects that affect the function and resilience of ecosystems.

## 5. Conclusions

This study demonstrates that psychoactive pharmaceuticals can induce compound-specific physiological effects in *L. minor*, with the relative growth rate identified as the most sensitive endpoint. Toxicity varied substantially among the compounds, with topiramate, clonazepam, and risperidone exhibiting higher potency. Despite measurable physiological effects, photosystem II efficiency remained largely unaffected, indicating that sublethal impacts are primarily associated with metabolic processes rather than direct PSII impairment by the psychoactive pharmaceuticals used in this study. Most compounds showed negligible abiotic degradation, confirming their persistence in aquatic systems, whereas sertraline and fluoxetine exhibited higher degradation rates than the other compounds. *Lemna minor* displayed relevant phytoremediation capacity, achieving high removal efficiencies for several compounds; however, this performance was consistently reduced under mixture conditions. Probabilistic risk assessment revealed a high cumulative ecological risk, with risperidone contributing most significantly to the mixture toxicity. These findings highlight that psychoactive pharmaceuticals represent a class of contaminants capable of simultaneously affecting plant physiology, persisting in aquatic environments, and contributing to mixture-driven ecological risks. Overall, the integration of ecotoxicological endpoints, removal efficiency, and risk assessment provides a robust framework for evaluating the environmental implications of psychoactive pharmaceuticals and supports the application of plant-based systems as complementary strategies in wastewater treatment.

## Figures and Tables

**Table 1:** Ecotoxicological parameters derived from concentration–response modeling of *Lemna minor* exposed to psychoactive pharmaceuticals. The values included NOEC, LOEC, EC_10_, EC_20_, and EC_50_ (ng L^−1^), along with model fit metrics (R^2^) and residual diagnostics.

Compound	NOEC	LOEC	EC10	EC20	EC50	Hill Slope	Max. Inhibition (%)	R^2^	Residual Normality	95% CI (EC50)	Model Performance
Citalopram	n.e.	25,000	57,593	67,892	89,941	−4.929	60	0.88	yes	78,220–102,171	Acceptable fit (steep slope)
Sertraline	50	75	n.e.	n.e.	n.e.	n.e.	n.e.	n.e.	n.e.	n.e.	No reliable fit
Fluoxetine	50	100	64.33	217.8	1751	−0.665	100	0.76	no	1161–2796	Acceptable fit (residual deviation)
Alprazolam	n.e.	3000	3836	22,630	465,351	−0.4585	100	0.81	yes	281,637–831,633	Acceptable fit (wide CI)
Clonazepam	n.e.	5	42.02	58.88	104.8	−2.405	70	0.92	yes	84.70–182.2	Good fit
Risperidone	40	60	4.50	15.93	138.5	−0.6041	100	0.92	yes	107.4–177.0	Good fit
Topiramate	n.e.	5	3.51	10.82	74.11	−0.7205	66	0.90	yes	47.94–176.3	Good fit

The no observed effect concentration (NOEC) and lowest observed effect concentration (LOEC) were determined using one-way ANOVA, followed by Dunnett’s test (*p* < 0.05). The EC_10_, EC_20_, and EC_50_ values (ng L^−1^) were estimated from the fitted models. The model performance was evaluated based on the goodness-of-fit (R^2^), residual diagnostics, and parameter confidence intervals, as described in Section 2.8. “n.e.” indicates parameters that could not be estimated due to inadequate model fit or the absence of a sigmoidal response.

**Table 3:** Toxicological contribution of psychoactive pharmaceuticals to cumulative mixture risk (msPAF_total_).

Acute Exposure Scenario							
Parameter	Citalopran	Sertraline	Fluoxetine	Alprazolan	Clonazepan	Risperidone	Topiramate
μTMoA	5.35	4.94	4.59	5.47	4.72	1.23	4.50
RISKTMoA	0.91	0.44	0.62	0.69	0.42	1.38	0.47
msPAF	0.86	0.65	0.70	0.76	0.56	0.90	0.57
Relative contribution (%)	28.89	0.93	6.90	4.45	16.40	66.51	19.08
msPAFtotal	1.00						
**Chronic exposure scenario**							
**Parameter**	**Citalopran**	**Sertraline**	**Fluoxetine**	**Alprazolan**	**Clonazepan**	**Risperidone**	**Topiramate**
μTMoA	3.70	3.94	3.59	4.47	3.71	0.21	3.50
RISKTMoA	1.32	0.56	0.79	0.84	0.54	7.24	0.62
msPAF	0.54	0.41	0.47	0.47	0.46	0.80	0.47
Relative contribution (%)	82.33	0.93	26.50	24.34	26.95	91.49	30.93
msPAFtotal	1.00						

μTMoA represents the position of each compound within the species sensitivity distribution, RISKTMoA corresponds to the compound-specific risk contribution, and msPAF indicates the potentially affected fraction of the population. The relative contribution (%) is not normalized to sum to 100%. The msPAF_total_ represents the cumulative risk of the mixture.

## Data Availability

The original contributions presented in this study are included in the article/Supplementary Materials. Further inquiries can be directed to the corresponding author.

## Supplementary Materials

The following supporting information can be downloaded at: https://www.mdpi.com/article/10.3390/toxics14050420/s1, Figure S1. Concentration–response curves for the relative growth rate (RGR, % of control) of *Lemna minor* exposed to psychoactive pharmaceuticals: (A) citalopram, (B) fluoxetine, (C) alprazolam, (D) clonazepam, (E) risperidone, and (F) topiramate. Table S1: Comparison between control and solvent control (DMSO) for physiological parameters of *Lemna minor*; Table S2: One-way ANOVA results for the effects of psychoactive pharmaceuticals on physiological parameters of *Lemna minor*; Table S3: Initial (T_0_) and final (T_7_) concentrations and abiotic degradation (%) of psychoactive pharmaceuticals in plant-free systems; Table S4: Ecotoxicological dataset and probabilistic risk assessment of psychoactive pharmaceuticals based on species sensitivity data and mode-of-action (TMoA) grouping.

## References

[1] Adeola A.O., Ore O.T., Fapohunda O., Adewole A.H., Akerele D.D., Akingboye A.S., Oloye F.F. (2022). Psychotropic Drugs of Emerging Concerns in Aquatic Systems: Ecotoxicology and Remediation Approaches. Chem. Afr..

[2] Du J., Zhang X., Li B., Huo S., Zhang J., Fu Y., Song M., Shao B., Li Y. (2024). The Hepatotoxicity of Hexafluoropropylene Oxide Trimer Acid Caused by Apoptosis via Endoplasmic Reticulum-Mitochondrial Crosstalk. Sci. Total Environ..

[3] Christensen A.M., Markussen B., Baun A., Halling-Sørensen B. (2009). Probabilistic Environmental Risk Characterization of Pharmaceuticals in Sewage Treatment Plant Discharges. Chemosphere.

[4] Cunha D.L., Mendes M.P., Marques M. (2019). Environmental Risk Assessment of Psychoactive Drugs in the Aquatic Environment. Environ. Sci. Pollut. Res..

[5] Gomes M.P., Gomes L.P. (2024). Tracking the Surge of Psychiatric Pharmaceuticals in Urban Rivers of Curitiba amidst and beyond the SARS-CoV-2 Pandemic. Sci. Total Environ..

[6] Gurke R., Rossmann J., Schubert S., Sandmann T., Rößler M., Oertel R., Fauler J. (2015). Development of a SPE-HPLC–MS/MS Method for the Determination of Most Prescribed Pharmaceuticals and Related Metabolites in Urban Sewage Samples. J. Chromatogr. B.

[7] Christensen A.M., Faaborg-Andersen S., Flemming I., Baun A. (2007). Mixture and Single-Substance Toxicity of Selective Serotonin Reuptake Inhibitors toward Algae and Crustaceans. Environ. Toxicol. Chem..

[8] Minguez L., Pedelucq J., Farcy E., Ballandonne C., Budzinski H., Halm-Lemeille M.-P. (2016). Toxicities of 48 Pharmaceuticals and Their Freshwater and Marine Environmental Assessment in Northwestern France. Environ. Sci. Pollut. Res..

[9] Henry T.B., Kwon J.-W., Armbrust K.L., Black M.C. (2004). Acute and Chronic Toxicity of Five Selective Serotonin Reuptake Inhibitors in Ceriodaphnia Dubia. Environ. Toxicol. Chem..

[10] Minguez L., Di Poi C., Farcy E., Ballandonne C., Benchouala A., Bojic C., Cossu-Leguille C., Costil K., Serpentini A., Lebel J.-M. (2014). Comparison of the Sensitivity of Seven Marine and Freshwater Bioassays as Regards Antidepressant Toxicity Assessment. Ecotoxicology.

[11] Brausch J.M., Connors K.A., Brooks B.W., Rand G.M. (2012). Human Pharmaceuticals in the Aquatic Environment: A Review of Recent Toxicological Studies and Considerations for Toxicity Testing. Reviews of Environmental Contamination and Toxicology.

[12] Aich U., Polverino G., Yazdan Parast F., Melo G.C., Tan H., Howells J., Nosrati R., Wong B.B.M. (2025). Long-term Effects of Widespread Pharmaceutical Pollution on Trade-offs between Behavioural, Life-history and Reproductive Traits in Fish. J. Anim. Ecol..

[13] Kalichak F., de Alcantara Barcellos H.H., Idalencio R., Koakoski G., Soares S.M., Pompermaier A., Rossini M., Barcellos L.J.G. (2019). Persistent and Transgenerational Effects of Risperidone in Zebrafish. Environ. Sci. Pollut. Res..

[14] Gomes T.B., Fernandes Sales Junior S., Saint’Pierre T.D., Correia F.V., Hauser-Davis R.A., Saggioro E.M. (2019). Sublethal Psychotropic Pharmaceutical Effects on the Model Organism Danio Rerio: Oxidative Stress and Metal Dishomeostasis. Ecotoxicol. Environ. Saf..

[15] Liu J.Y., Sayes C.M. (2024). Modeling Mixtures Interactions in Environmental Toxicology. Environ. Toxicol. Pharmacol..

[16] Gomes M.P. (2024). Climate Change and Aquatic Phytoremediation of Contaminants: Exploring the Future of Contaminant Removal. Phyton-Int. J. Exp. Bot..

[17] Goala M., Bachheti A., Kumar Arya A., Kumar V. (2025). A Review on the Role of Duckweed (*Lemna* spp.) in the Rejuvenation of Aquatic Bodies by Pollutant Remediation and Recovery of Valuable Resources. Environ. Monit. Assess..

[18] Paolacci S., Stejskal V., Jansen M.A.K. (2021). Estimation of the Potential of Lemna Minor for Effluent Remediation in Integrated Multi-Trophic Aquaculture Using Newly Developed Synthetic Aquaculture Wastewater. Aquac. Int..

[19] Sarkheil M., Safari O. (2020). Phytoremediation of Nutrients from Water by Aquatic Floating Duckweed (*Lemna minor*) in Rearing of African Cichlid (*Labidochromis lividus*) Fingerlings. Environ. Technol. Innov..

[20] Maldonado I., Moreno Terrazas E.G., Vilca F.Z. (2022). Application of Duckweed (*Lemna* sp.) and Water Fern (*Azolla* sp.) in the Removal of Pharmaceutical Residues in Water: State of Art Focus on Antibiotics. Sci. Total Environ..

[21] Formagini L., Ramirez J.Z.R., Corá V.R., Souza D.M. (2025). Psychotropic Pharmaceuticals in Aquatic Environments: Occurrence and Analytical Challenges. Sci. Total Environ..

[22] Chu S.P. (1942). The Influence of the Mineral Composition of the Medium on the Growth of Planktonic Algae. J. Ecol..

[23] Organization for Economic Co-operation and Development OECD (2006). Test No. 221: *Lemna* sp. Growth Inhibition Test. OECD Guidelines for the Testing of Chemicals.

[24] Liang J., Meng H., Zhou J., Yin Y., Zhen H., Zhao Y., Zhang K. (2021). Simultaneous Occurrence of Psychotropic Pharmaceuticals in Surface Water of the Megacity Shanghai and Implication for Their Ecotoxicological Risks. ACS ES&T Water.

[25] Metcalfe C.D., Chu S., Judt C., Li H., Oakes K.D., Servos M.R., Andrews D.M. (2010). Antidepressants and Their Metabolites in Municipal Wastewater, and Downstream Exposure in an Urban Watershed. Environ. Toxicol. Chem..

[26] Cunha D.L., de Araujo F.G., Marques M. (2017). Psychoactive Drugs: Occurrence in Aquatic Environment, Analytical Methods, and Ecotoxicity—A Review. Environ. Sci. Pollut. Res..

[27] Kitajima M., Butler W.L.W. (1975). Quenching of Chlorophyll Fluorescence and Primary Photochemistry in Chloroplasts by Dibromothymoquinone. Biochim. Biophys. Acta.

[28] USEPA (2001). Risk Assessment Guidance for Superfund: Volume III—Part A, Process for Conducting Probabilistic Risk Assessment.

[29] Rico A., de Oliveira R., de Souza Nunes G.S., Rizzi C., Villa S., López-Heras I., Vighi M., Waichman A.V. (2021). Pharmaceuticals and Other Urban Contaminants Threaten Amazonian Freshwater Ecosystems. Environ. Int..

[30] Posthuma L., Suter G.W., Traas T.P. (2001). Species Sensitivity Distributions in Ecotoxicology.

[31] Zhu X., Luo T., Wang D., Zhao Y., Jin Y., Yang G. (2023). The Occurrence of Typical Psychotropic Drugs in the Aquatic Environments and Their Potential Toxicity to Aquatic Organisms—A Review. Sci. Total Environ..

[32] Khasawneh O.F.S., Palaniandy P. (2021). Occurrence and Removal of Pharmaceuticals in Wastewater Treatment Plants. Process Saf. Environ. Prot..

[33] Dodgson S.J., Shank R.P., Maryanoff B.E. (2000). Topiramate as an Inhibitor of Carbonic Anhydrase Isoenzymes. Epilepsia.

[34] Erland L.A.E., Turi C.E., Saxena P.K. (2019). Serotonin in Plants: Origin, Functions, and Implications. Serotonin.

[35] Feijão E., Cruz de Carvalho R., Duarte I.A., Matos A.R., Cabrita M.T., Utkin A.B., Caçador I., Marques J.C., Novais S.C., Lemos M.F.L. (2022). Fluoxetine Induces Photochemistry-Derived Oxidative Stress on *Ulva lactuca*. Front. Environ. Sci..

[36] Ankley P.J., Stewart J., Nash M., Schumann P.G., Flynn K., Villeneuve D.L. (2025). Sertraline Hydrochloride Exposure Leads to Reactive Oxygen Species Burst in Model Microalgae Species (*Raphidocelis subcapitata*). Environ. Sci. Technol..

[37] Gomes M.P., Brito J.C.M., Rocha D.C., Navarro-Silva M.A., Juneau P. (2020). Individual and Combined Effects of Amoxicillin, Enrofloxacin, and Oxytetracycline on *Lemna minor* Physiology. Ecotoxicol. Environ. Saf..

[38] Gomes M.P., Malinoski L., Maranho L.T., Carneiro D.N.M., Richardi V.S., Martinez M.G. (2026). Microbiota Modulate Metformin Phytoremediation and Stress Responses in Lemna Minor. J. Hazard. Mater..

[39] Ma Z.S. (2024). Species Specificity and Specificity Diversity (SSD) Framework: A Novel Method for Detecting the Unique and Enriched Species Associated with Disease by Leveraging the Microbiome Heterogeneity. BMC Biol..

[40] Lin W., Zhao B., Ping S., Zhang X., Ji Y., Ren Y. (2022). Ultraviolet Oxidative Degradation of Typical Antidepressants: Pathway, Product Toxicity, and DFT Theoretical Calculation. Chemosphere.

[41] Lam M.W., Young C.J., Mabury S.A. (2005). Aqueous Photochemical Reaction Kinetics and Transformations of Fluoxetine. Environ. Sci. Technol..

[42] Couto E., Assemany P.P., Assis Carneiro G.C., Ferreira Soares D.C. (2022). The Potential of Algae and Aquatic Macrophytes in the Pharmaceutical and Personal Care Products (PPCPs) Environmental Removal: A Review. Chemosphere.

[43] Chaurasia S. (2022). Role of Macrophytes: A Review. Adv. Zool. Bot..

[44] De M., Roy C., Medda S., Roy S., Dey S.R. (2019). Diverse Role of Macrophytes in Aquatic Ecosystems: A Brief Review. Int. J. Exp. Res. Rev..

