# Supplementary material for: Ecotoxicological Effects of Psychoactive Pharmaceuticals in Lemna minor: Phytoremediation Potential and Mixture Risk Assessment
Source: Toxics. 2026 May 12;14(5):420. doi: 10.3390/toxics14050420 (PMC13211527; doi:10.3390/toxics14050420)
Supplement: Supplementary file 1 [file toxics-14-00420-s001.zip › toxics-4292209-supplementary.pdf]

Table S1. Comparison between control and solvent control (DMSO) for physiological parameters of *Lemna minor*.

Treatment	RGR (g g^{-1} d^{-1})	Chlorophyll (SPAD)	Fv/Fm
Control	0.082 ± 0.004	15.8 ± 0.7	0.78 ± 0.02
DMSO (0.1% v/v)	0.080 ± 0.005	15.5 ± 0.8	0.77 ± 0.02
p-value	0.421	0.337	0.515

Values are presented as mean ± standard deviation (n = 3). Differences between treatments were evaluated using Student's *t*-test.

Table S2. One-way ANOVA results for the effects of psychoactive pharmaceuticals on physiological parameters of *Lemna minor*.

Compound	DF	RGR (F)	RGR (p)	Chlorophyll (F)	Chlorophyll (p)	Fv/Fm (F)	Fv/Fm (p)
Citalopram	9	90.038	<0.0001*	1.977	0.098	3.266	0.013*
Sertraline	9	47.065	<0.0001*	3.466	0.0099*	5.492	0.0007*
Fluoxetine	9	27.986	<0.0001*	1.347	0.2754	10.635	<0.0001*
Alprazolam	9	188.187	<0.0001*	6.877	0.0002*	2.365	0.052
Clonazepam	9	188.513	<0.0001*	5.947	0.0008*	3.613	0.0112*
Risperidone	9	79.619	<0.0001*	1.921	0.1221	1.848	0.1359
Topiramate	9	107.708	<0.0001*	1.905	0.1102	6.720	0.0002*

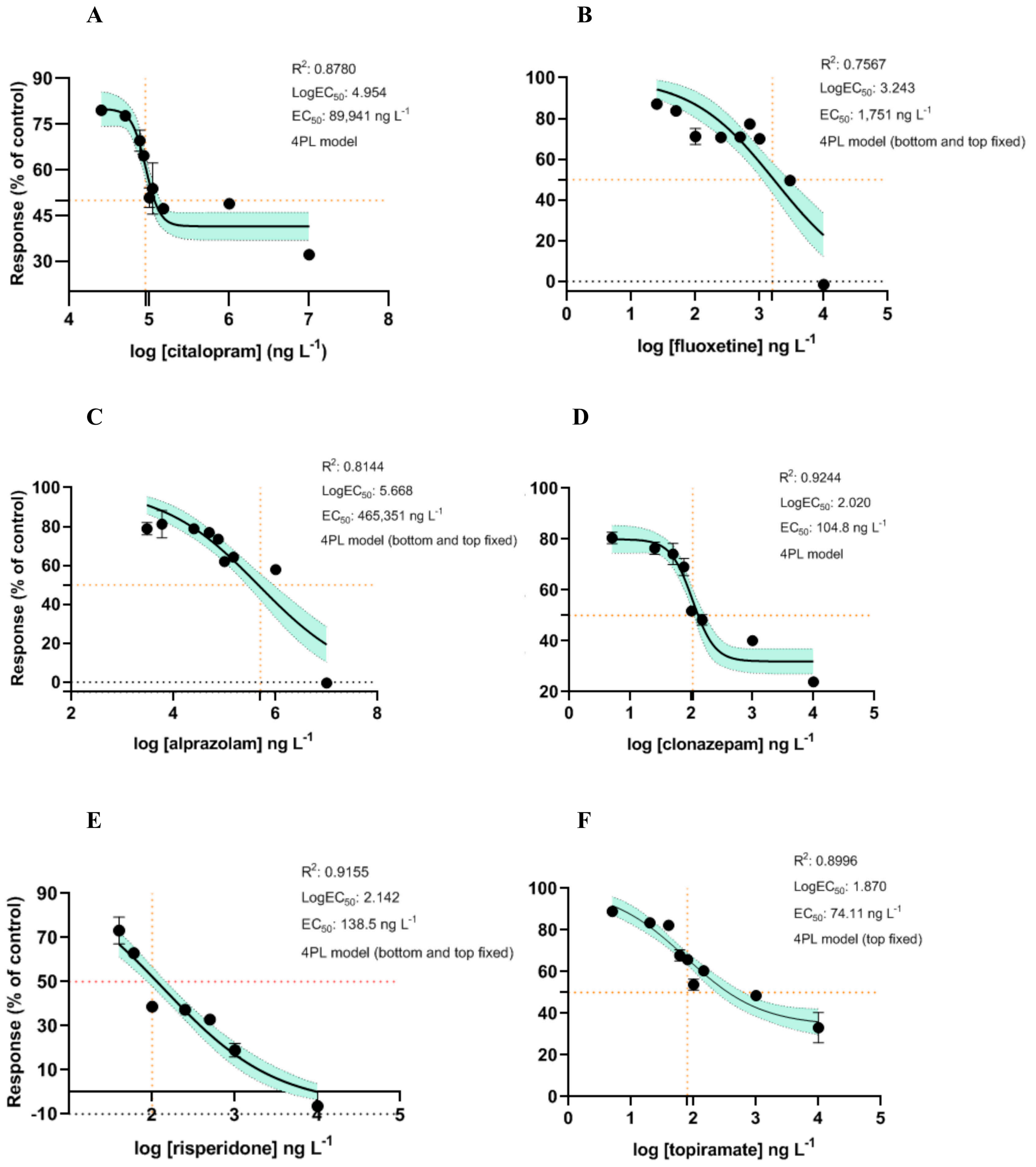

Figure S1. Concentration–response curves for the relative growth rate (RGR, % of control) of *Lemna minor* exposed to psychoactive pharmaceuticals: (A) citalopram, (B) fluoxetine, (C) alprazolam, (D) clonazepam, (E) risperidone, and (F) topiramate. Data points represent mean ± standard deviation (n = 3). Solid lines represent the fitted four-parameter logistic (4PL) models, with constraints applied when necessary to ensure model stability and fit quality, and shaded areas represent the 95% confidence intervals. Orange dashed lines denote the estimated EC_{50} values (horizontal: 50% effect; vertical: corresponding log concentration). Model fit statistics (R^2), $LogEC_{50}$, and EC_{50} values (ng L^{-1}) are shown within each panel.

Table S3. Initial (T_0) and final (T_7) concentrations and abiotic degradation (%) of psychoactive pharmaceuticals in plant-free systems.

Nominal concentration (ng L-1)	T_0 (ng L^{-1})	T_7 (ng L^{-1})	Abiotic degradation (%)	p<0.05
**Control**	0.00 ± 0.00	0.00 ± 0.00	nd	-
**Citalopram**				
25,000	24,966.67 ± 321.46	24,900.00 ± 264.58	0.27 ± 0.23	a
50,000	49,933.33 ± 862.17	49,900.00 ± 818.54	0.07 ± 0.11	a
76,000	76,133.33 ± 1250.33	76,066.67 ± 1201.39	0.09 ± 0.08	a
85,000	84,933.33 ± 1656.30	84,833.33 ± 1656.30	0.12 ± 0.00	a
100,000	100,033.33 ± 1222.02	99,933.33 ± 1222.02	0.10 ± 0.00	a
110,000	110,333.33 ± 2516.61	109,666.67 ± 2081.67	0.60 ± 0.52	a
150,000	150,000.00 ± 3000.00	149,000.00 ± 3000.00	0.67 ± 0.01	a
1,000,000	1,011,000.00 ± 8544.00	1,009,666.67 ± 8020.81	0.13 ± 0.06	a
10,000,000	10,050,000.00 ± 88881.94	10,023,333.33 ± 85049.01	0.27 ± 0.06	a
76,000 (Mix)	76,133.33 ± 1250.33	76033.33 ± 1201.39	0.13 ± 0.05	*
**Sertraline**				
50	50.20 ± 0.62	28.50 ± 0.46	43.23 ± 0.18	bc
75	75.03 ± 1.20	42.70 ± 0.70	43.09 ± 0.04	bc
100	100.37 ± 0.72	57.23 ± 0.75	42.98 ± 0.37	c
125	125.00 ± 3.00	71.23 ± 1.75	43.01 ± 0.03	c
155	155.00 ± 4.00	88.33 ± 2.30	43.01 ± 0.03	c
250	250.67 ± 4.51	142.33 ± 3.06	43.22 ± 0.20	bc
500	503.33 ± 8.50	284.00 ± 5.00	43.58 ± 0.04	ab
1,000	1,005.00 ± 10.00	565.67 ± 6.03	43.72 ± 0.06	a
10,000	10,023.33 ± 165.63	5,653.33 ± 90.18	43.60 ± 0.03	ab
155 (Mix)	155.00 ± 4.00	90.20 ± 2.10	41.81 ± 0.42*	*
**Fluoxetine**				
25	25.07 ± 0.31	19.20 ± 0.36	23.41 ± 0.51	bcd
50	50.17 ± 0.70	38.33 ± 0.55	23.59 ± 0.04	bcd
100	100.30 ± 0.56	77.07 ± 0.67	23.16 ± 0.24	d
250	250.67 ± 4.51	191.67 ± 3.51	23.54 ± 0.04	cd
500	503.33 ± 8.50	385.00 ± 6.56	23.51 ± 0.13	bcd
706	706.33 ± 12.01	538.67 ± 9.02	23.74 ± 0.15	abc
1,000	1,003.67 ± 8.50	766.67 ± 6.51	23.61 ± 0.01	abcd
3,000	3,010.00 ± 70.00	2,293.33 ± 55.08	23.81 ± 0.11	ab
10,000	10,003.33 ± 138.68	7,600.00 ± 111.36	24.03 ± 0.10	a
706 (Mix)	706.33 ± 12.01	552.00 ± 8.50	21.85 ± 0.31	*
**Alprazolam**				
3,000	3,003.33 ± 47.26	2,943.33 ± 47.26	2.00 ± 0.03	a
59,000	59,000.00 ± 1081.66	57,800.00 ± 1866.54	2.03 ± 0.04	a
25,000	25,133.33 ± 450.92	24,633.33 ± 450.92	1.99 ± 0.04	a
50,000	49,933.33 ± 850.49	48,933.33 ± 850.49	2.00 ± 0.03	a
75,000	75,066.67 ± 1150.36	73,566.67 ± 1150.36	2.00 ± 0.03	a
100,000	100,033.33 ± 1150.36	98,033.33 ± 1150.36	2.00 ± 0.02	a
150,000	149,666.67 ± 2516.61	146,666.67 ± 2516.61	2.00 ± 0.03	a
1,000,000	1,008,000.00 ± 8888.19	988,000.00 ± 8888.19	1.98 ± 0.02	a
10,000,000	10,003,333.33 ± 138684.29	9,803,333.33 ± 138684.29	2.00 ± 0.03	a

5,900 (Mix)	5,900.00 ± 120.00	5,785.00 ± 115.00	1.95 ± 0.03	-
**Clonazepam**				
5	5.00 ± 0.10	4.95 ± 0.10	0.93 ± 0.10	ab
25	25.10 ± 0.70	24.90 ± 0.70	0.80 ± 0.02	ab
50	50.03 ± 1.15	49.63 ± 1.15	0.80 ± 0.02	ab
75	75.17 ± 1.65	74.57 ± 1.65	0.80 ± 0.02	ab
100	100.20 ± 1.10	99.40 ± 1.10	0.80 ± 0.01	ab
150	150.00 ± 3.00	148.00 ± 3.00	1.33 ± 0.03	a
1,000	1,005.00 ± 10.00	997.33 ± 9.50	0.76 ± 0.05	b
10,000	10,016.67 ± 202.07	9,936.67 ± 202.07	0.80 ± 0.02	ab
100 (Mix)	100.20 ± 1.10	99.10 ± 1.10	1.10 ± 0.04	*
**Risperidone**				
40	40.00 ± 0.36	39.13 ± 0.31	2.17 ± 0.13	a
60	60.10 ± 0.82	58.90 ± 0.82	2.00 ± 0.03	a
100	100.03 ± 0.86	97.93 ± 0.86	2.10 ± 0.02	a
250	250.00 ± 2.00	245.00 ± 2.00	2.00 ± 0.02	a
500	502.00 ± 4.00	492.00 ± 4.00	1.99 ± 0.02	a
1,000	1,007.67 ± 8.74	987.67 ± 8.74	1.98 ± 0.02	a
10,000	10,040.00 ± 157.16	9,840.00 ± 157.16	1.99 ± 0.03	a
50 (Mix)	50.00 ± 0.60	49.00 ± 0.60	2.00 ± 0.03	-
**Topiramate**				
5	5.00 ± 0.10	4.97 ± 0.10	0.67 ± 0.12	ab
20	20.07 ± 0.65	19.97 ± 0.65	0.50 ± 0.02	c
40	40.10 ± 1.10	39.90 ± 1.10	0.50 ± 0.01	c
60	60.27 ± 1.20	59.87 ± 1.20	0.66 ± 0.01	ab
80	80.27 ± 1.65	79.77 ± 1.65	0.62 ± 0.01	ab
100	100.30 ± 0.62	99.70 ± 0.62	0.60 ± 0.00	bc
145	145.33 ± 3.51	144.33 ± 3.51	0.69 ± 0.02	a
1,000	1,005.00 ± 10.00	999.00 ± 10.00	0.60 ± 0.01	bc
10,000	10,023.33 ± 165.63	9,963.33 ± 165.63	0.60 ± 0.01	bc
145 (Mix)	145.33 ± 3.51	143.90 ± 3.51	0.98 ± 0.02	*

*Indicates significant differences between isolated and mixture treatments (Student's *t*-test, $p < 0.05$).
